# Supplementary material for: Genetically Modified Ferritin Nanoparticles with Bone-Targeting Peptides for Bone Imaging
Source: Int J Mol Sci. 2021 May 3;22(9):4854. doi: 10.3390/ijms22094854 (PMC8125493; doi:10.3390/ijms22094854)
Supplement: Supplementary file 1 [file ijms-22-04854-s001.zip › ijms-1201072-supplementary.pdf]

Supplementary information

Genetically modified ferritin nanoparticles with bone-targeting peptides for bone imaging

Jong-Won Kim[1], Kyung Kwan Lee[1,3], Kyoung Woo Park[1,2],

Moonil Kim[1,2,*] and Chang-Soo Lee[1,2,]**

[1] Bionanotechnology Research Center, Korea Research Institute of Bioscience & Biotechnology (KRIBB), Daejeon 34141, South Korea

[2] Department of Biotechnology, University of Science & Technology (UST), Daejeon 34113, South Korea

[3] Department of Life and Nanopharmaceutical Science, College of Pharmacy, Kyung Hee University, Seoul 02447, South Korea

*Correspondence: cslee@kribb.re.kr; +82-42-879-8446

* Co-corresponding author

Fabrication of calcium deficient hydroxyapatite (CDHA) scaffolds

α-tricalcium phosphate (α-TCP) paste with proper rheological characteristics for stacking a stable three-dimensional (3D) structure through a 3D printing system was formulated by mixing ground powder with 1 wt% solution of hydroxypropyl methyl cellulose prepared in 30% ethanol. The powder to liquid ratio was set to 1.67 for effective extrusion. α-TCP scaffolds were fabricated using a paste extruding deposition (PED) system, which is a representative 3D printing system, and were dried at 37 °C for 24 h before cementation. Pure calcium deficient hydroxyapatite (CDHA) scaffolds were prepared by hydrolyzing of α-TCP scaffolds in PBS solution for 24 h and then washed with deionized water several times and dried completely at room temperature for three days prior to further experiments.

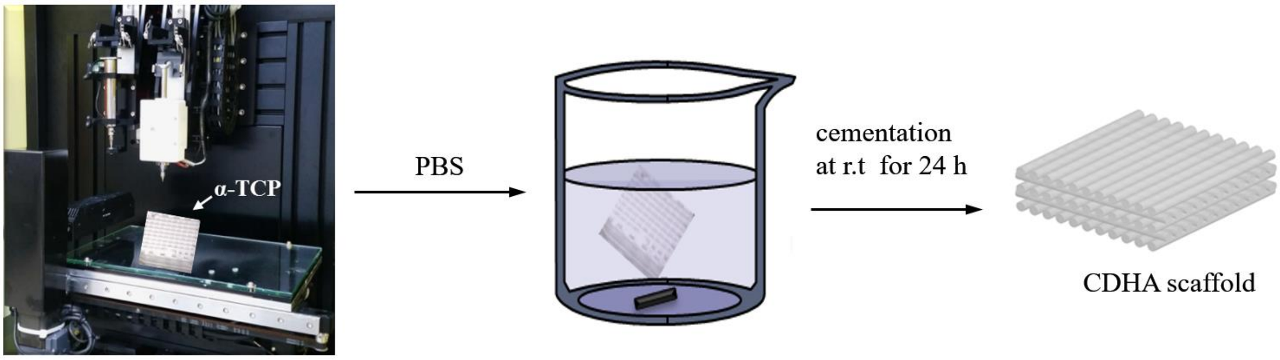

Figure S1. Schematic diagram of CDHA scaffold fabrication.

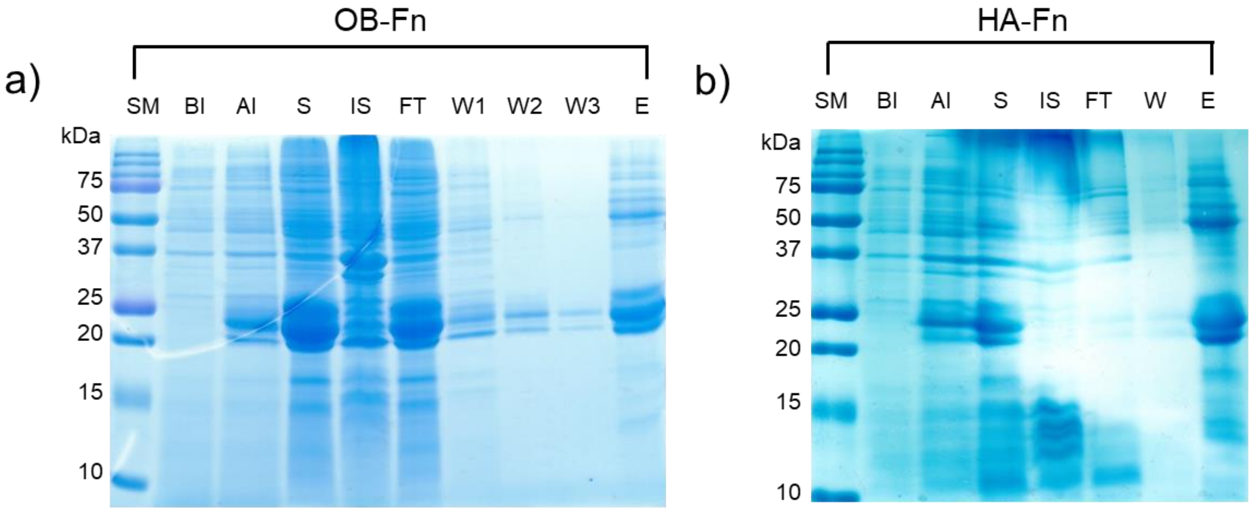

Figure S2. SDS-PAGE analysis of (a) OB-Fn and (b) HA-Fn nanoparticles using 12% SDS-PAGE gel. BI : cells before induction using IPTG; AI : harvested cells after induction using IPTG; S : soluble supernatant after cell lysis; IS : insoluble pellet after cell lysis; FT : flow-through during purification using Ni-NTA resin; W : washing; E : elution.
